# WHO’s Robson platform for data-sharing on caesarean section rates

**DOI:** 10.2471/BLT.21.287742

**Published:** 2022-04-04

**Authors:** Newton Opiyo, Maria Regina Torloni, Michael Robson, Lars Ladfors, Karima Gholbzouri, Justina Kacerauskiene, Rafael Vila-Candel, Joerg Kessler, Miha Lucovnik, Ana Pilar Betrán

**Affiliations:** a UNDP/UNFPA/UNICEF/WHO/World Bank Special Programme of Research, Development and Research Training in Human Reproduction (HRP), Department of Reproductive Health and Research, World Health Organization, Avenue Appia 20, 1211 Geneva 27, Switzerland.; b Evidence Based Healthcare Postgraduate Program, São Paulo Federal University, São Paulo, Brazil.; c National Maternity Hospital, Dublin, Ireland.; d Department of Obstetrics and Gynaecology, Sahlgrenska University Hospital, Gothenburg, Sweden.; e Department of Health Promotion, World Health Organization Regional Office for the Eastern Mediterranean, Cairo, Egypt.; f Lithuanian University of Health Sciences, Kaunas, Lithuania.; g Hospital de La Ribera, Valencia, Spain.; h Department of Obstetrics and Gynaecology, Haukeland University Hospital, Bergen, Norway.; i Department of Perinatology, University Medical Centre Ljubljana, Ljubljana, Slovenia.

Optimizing caesarean section use is a global health priority, given the maternal and perinatal morbidity and mortality associated with caesarean underuse and overuse.^1^^,^^2^ Monitoring caesarean section rates is important to understand trends, identify inequities in their use, and develop and implement strategies to optimize their use. However, the lack of an internationally accepted classification system has hindered routine global monitoring of caesarean section rates. In addition, monitoring overall aggregate caesarean section rates is not sufficient. Finer disaggregated data are needed to characterize and pinpoint obstetric subgroups driving caesarean section rates and to support appropriate intervention targeted at the appropriate groups which most contribute to the overall caesarean section rate.

In 2015, the World Health Organization (WHO) recommended the 10-group classification system: the Robson classification, as a global standard for assessing, monitoring and comparing caesarean section rates within and between maternity units worldwide.^3^ The system classifies all women at admission for birth into 10 groups based on basic obstetric characteristics that are routinely collected in maternity units worldwide (parity and previous caesarean sections, number of fetuses, gestational age, fetal presentation and lie, and onset of labour). The structure of the classification allows users to better analyse and understand labour events, clinical practices, indications, outcomes and significant epidemiological factors including case mix. The classification serves as a common language necessary to bring health practitioners together in a constructive debate about clinical practices in relation to caesarean sections. A 2018 systematic review of six studies showed that implementation of the Robson classification may be associated with reduced caesarean section rates.^4^

The Robson classification has gained wide acceptance in a diverse range of health-care, research and policy-making settings worldwide,^5^^,^^6^ and its widespread adoption presents an opportunity to monitor and compare caesarean section rates and perinatal outcomes on a much larger scale using a similar and standard method. In 2017, to assist health-care facilities in adopting and using the Robson classification, WHO developed guidance for its use, implementation and interpretation, including standardization of terms and definitions.^7^

WHO announces another tool to facilitate the use of the Robson classification, the Robson platform.^8^ This global, free, interactive platform is a place where individual maternity units worldwide can upload and share their hospital-level caesarean and associated perinatal outcome data using the Robson classification system. The data are available openly and updated in real time as soon as facilities upload new data.

The data from the platform can be used for multiple purposes: (i) monitoring and comparing trends of caesarean section rates and associated outcomes across different settings; (ii) identification of groups of women which most contribute to overall caesarean section rates; (iii) evaluation of policies and interventions to optimize caesarean section use; (iv) assessment of the quality of care and obstetric practices by analysing outcomes across diverse settings; and (v) assessment of the quality of obstetric data including the proportion of unclassified women due to missing information.^7^

**Fig. 1:**
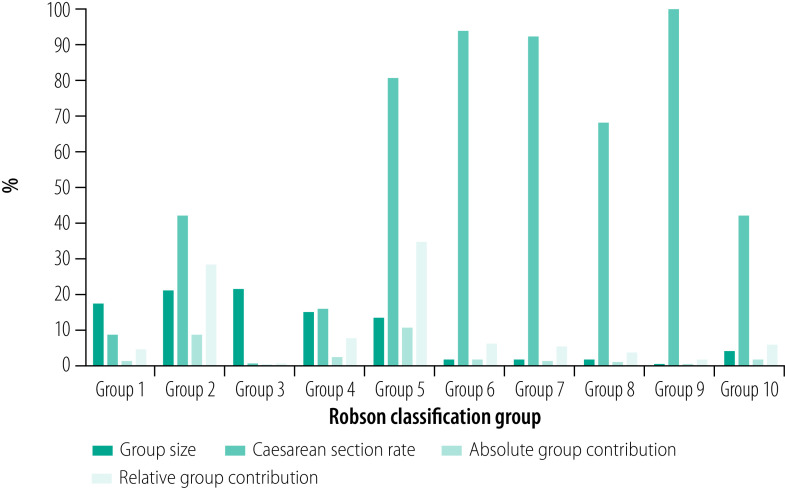
Caesarean section rates by Robson classification, the National Maternity Hospital, Ireland, 2020

Notes: The system classifies all women at admission for birth into 10 groups based on basic obstetric characteristics (parity and previous caesarean sections, number of fetuses, gestational age, fetal presentation and lie, and onset of labour). Group size is number of women in the group / total number of women delivered in the setting x 100. Caesarean section rate is number of caesarean sections in the group / total number of women in the group x 100. Absolute group contribution is number of caesarean sections in the group / total number of women delivered in the setting x 100. Relative group contribution is number of caesarean sections in the group / total number of caesarean sections in the setting x 100.

**Fig. 2:**
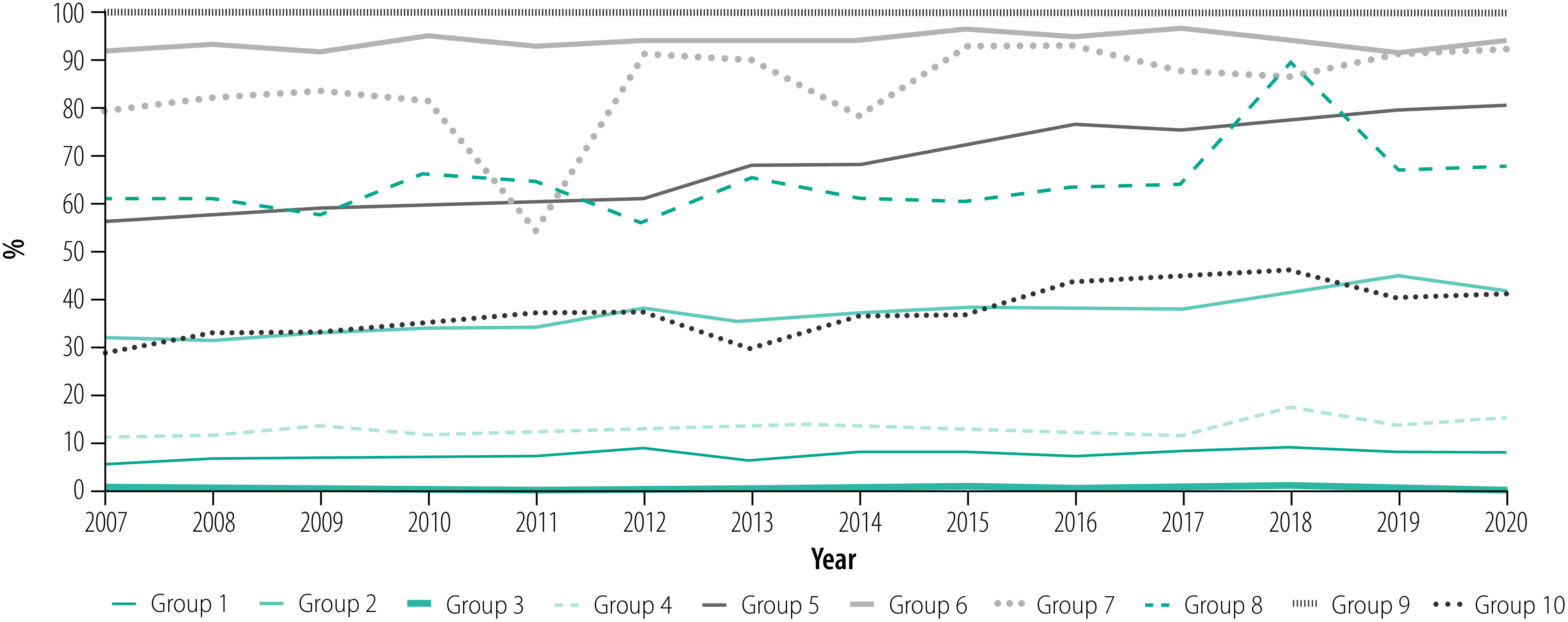
Trends in caesarean section rates by Robson group, the National Maternity Hospital, Ireland, 2007–2020

Notes: The system classifies all women at admission for birth into 10 groups based on basic obstetric characteristics that are routinely collected in maternity units worldwide (parity and previous caesarean sections, number of fetuses, gestational age, fetal presentation and lie, and onset of labour). Some caution is needed in interpreting these changes without knowing how the sizes of the groups have changed over the same period. In addition, other perinatal outcomes need to be included as well as significant epidemiological variables in the index population. The final interpretation of caesarean section rates can only be made when all these factors are included.

The platform provides easy access to data on caesarean section rates and associated perinatal outcomes and clinical processes in maternity units worldwide in the standard format of the Robson classification, allowing monitoring and comparison of caesarean section rates across maternity units and countries, and over time. The platform also allows users to create customized charts and graphs to visualize caesarean section data quickly and effortlessly at specific time points, or over time for the whole obstetric population or per individual Robson groups (Fig. 1 and Fig. 2). This feature of the platform allows users from different maternity units around the globe to engage in data-driven discussions and share experiences and clinical protocols or practices that may be relevant to optimize caesarean section use and outcomes.

In the Robson platform, users can view caesarean section rates for each health facility according to specific criteria of interest (for example, year or Robson group). Users will also be able to share data on clinical processes (such as definitions of spontaneous labour, induced labour and birth) that will enable a deeper understanding of differences in caesarean sections and perinatal outcomes across maternity units. The platform is dynamic and will undergo continuous improvement, with additional features added according to user needs and feedback.

When assessing and interpreting caesarean section rates and perinatal outcomes using the Robson classification, users are encouraged to consider other factors that are not provided in the platform but can have a significant impact on results. Health-system factors (models of care, payment systems for health providers and facilities, staffing or resource availability) and clinical processes that vary between maternity units (diagnosis of labour, fetal distress, management of dystocia, electronic fetal monitoring or indications of caesarean section) may impact caesarean section rates and outcomes.^9^

The platform can help to standardize routine audit of caesarean section rates and outcomes, simplify comparisons and quickly identify obstetric subgroups driving caesarean section rates. Enhanced understanding of the drivers of caesarean section trends can help users to develop more tailored and effective interventions for their setting. Embedding the Robson classification system into routine maternity data collection can motivate facilities to improve the quality of their obstetric data.

Caesarean section use is increasing worldwide; its use is unequal in low- and middle-income countries, and its underuse and overuse is associated with adverse outcomes. Therefore, using tools such as the Robson classification system is a priority for the health community. We hope that the platform will help build evidence to inform tailored, data-driven policies and actions to optimize caesarean use.
